# Human rights, vulnerability, and critical reflection on HIV/AIDS prevention in the syndemic context

**DOI:** 10.1590/0102-311XEN186423

**Published:** 2023-12-08

**Authors:** Vera Silvia Facciolla Paiva, José Ricardo de Carvalho Mesquita Ayres

**Affiliations:** 1 Instituto de Psicologia, Universidade de São Paulo, São Paulo, Brasil.; 2 Faculdade de Medicina, Universidade de São Paulo, São Paulo, Brasil.

The article by Grangeiro et al. ^1^ is very timely to wake us up from a lethargy that, in another context, has been described as the “trivialization of the epidemic” ^2^, i.e., the tendency to settle into a situation and cool down response capacities. If, at the end of the 1990s, the trivialization expressed the effect of the change in the sociodemographic profile of the most affected, when the AIDS epidemic reached peripheral populations, with less influence on public opinion and less organization to define policies, today’s trivialization represents what we could call a “fractional universalization”, as paradoxical as this expression may seem.

Universalization since, as pointed out in the article, the predominant proposal of “combination prevention” has often been translated, along the lines of neoliberal ideology, as a “package” of preventive options to be “used” according to the interest and convenience of each individual. In this sense of universality, each individual would have access to any of the available preventive technologies.

In fact, as the article also indicates, this proposition hides interconnected and underlying aspects of a supply of resources that is only apparently equitable and also only apparently equivalent in its practical implications. It conceals a totality, a context - historical, economic, political, and cultural - in which the processes by which prevention technologies are conceived, produced, and distributed are interrelated. The supposedly spontaneous supply and use of these technologies configure, uncritically, a policy of response to the HIV/AIDS epidemic that should be debated and constructed in the public space as a common good, subsidized by scientific evidence and guided by an ethical-political examination. Therefore, it is fractional.

Then, we are trivializing it since we accept, based on pseudoscientific argumentation, that technologies are neutral, ahistorical, and apolitical. Since we disregard the economic interests that pressure the dispute for the incorporation of technologies in health policies. Since we treat collectives as homogeneous masses of individuals and individuals as isolated and independent (cognitive, behavioral) (id)entities. Since we make risk and vulnerability synonymous with the same scientific operation: probability calculation.

Immersed in this lethargy, trivializing epidemics such as HIV, we continue to believe that the evident limit found in the purpose of controlling this epidemic, as well as others that will come in the context of the environmental crisis we are experiencing, will only be an expression of a quantitative deficit. Of course, scale is also a challenge, especially if we remember how many people are still excluded from access to basic prevention resources, but the issue seems to be about quantity: it is qualitative, above all, conceptually and politically.

The authors take a step towards this reflection when they discuss, for example, that the lack of investment and prioritization of strategies to cope with the HIV/AIDS epidemic have weakened the results of prevention policies aimed at adolescents and young people. They point out that disregarding the “social determinants” of the epidemic can lead to greater exposure to infection and, at the same time, coproduce an insufficient use of preventive resources necessary for prevention. For this reason, they advocate that intersectional analyses of determinants such as age, gender, race/ethnicity, among others, be adopted as guidelines for prevention policies, replacing the universal “core package” with the diversified and contextualized offer of preventive resources.

We also suggest other complementary moves in this direction.

First, it is necessary to retranslate the collective as the common, to understand how this common is constructed with people in interaction in the various contexts in which they find themselves, in the scenes of daily life in which they expose themselves (or are exposed) to sexually transmitted infections (STI).

Emphasizing that people only participate in the construction of the common when they assume themselves as subject of rights is necessary. Acknowledging this condition of shared citizenship in the concrete contexts in which it is conformed will allow us to understand and transform the articulated set of conditions that make us - adults and adolescents - vulnerable. Vulnerability, which is not synonymous with risk, despite being often expressed in it. Vulnerability, which is the systematic non-recognition of the other in his/her needs and potentialities, which is the absence of promotion, protection, and fulfilment of human rights ^3^.

Second, another necessary movement to radically shake up trivialization will be to overcome notions of the individual resulting from ideologies and religiosities circulating in all media, which take advantage of the absence of specific training for prevention. Interesting and productive theories, techniques, and psycho-educational practices for prevention have dialogued with the principles of the Brazilian Unified National Health System (SUS), developing literacy practices inherited from Freire’s pedagogy of the oppressed and of hope and stimulated the participation of adolescents and young people as subjects of sexual rights, part of comprehensive health care ^4^.

In their analysis, the authors highlight the need to provide information on the methods associated with reducing the incidence of HIV, those that have longer-lasting effects, or those that offer greater convenience and facilitate risk management since they are distant from “the sex scene”. They highlight the limits of the conceptions that continue to reduce the individual to a “consumer” of pre-formatted inputs and services without their participation, which have become more prevalent in contexts of neglect of the training of new professionals and persecution of public policies for the promotion of sexual health and perspectives based on human rights. They emphasize the need to acquire “skills to deal with the epidemic and prevention” - a perspective prevalent in the international literature, but without detailing what other preventive skills are needed.

In fact, as we have observed in the most recent experiences that we continue to develop in the field of the STI prevention with young people, in the context of the response to COVID-19, mpox, and the pandemic of mental health events (the one that most concerns community and school leaders in the territories where we are thinking about prevention with adolescents) ^5^, if we do not question the notions of the individual as a biological-behavioral unit, young people continue to reproduce prevention proposals similar to individualizing preaching and moral demand for results, even when carried out with their participation.

Rescuing is necessary always and each time - with young people and prevention agents - the person as a being always in interaction in and with their contexts, in the territories in which they live or circulate in their daily study, work, and leisure, understanding the current context of syndemic implied in the local scenario of inequalities. In our experience with prevention, the most productive and integrative core skill of all the others has been the codification of the dynamics of the exposure scene, implicated in the territorial scenario - with locally specific sociocultural characteristics and programmatic conditions in the SUS and Unified Social Assistance System (SUAS). We aim to educate young people and with young people about the structuring of each exposure scene and how to embody prevention in each scene they live.

Without this specific literacy work, “biological-behavioral individuals” continue to be held responsible for the success or failure of prevention, even if they eventually identify phenomena such as stigma and discrimination associated with sexuality that need to be overcome or inequality informed by the intersectional analysis of social determinants.

Finally, there is a point to be made to conclude these comments. Although we agree with the authors about the importance of pre-exposure prophylaxis (PrEP) as an effective input for the prevention of HIV infection, especially if we think about the use of drugs with long-lasting effects, it seems to us that its valorization by distance from the sexual scene can configure a dangerous trap, which the tradition of prevention guided by the principles of human rights has sought to avoid. Let us think of PrEP not as something detached from the sex scene. Rather, let us seek to explore how it can participate in sexual encounters that are more protected and protective of all people. We cannot risk failing to think collectively about how to protect sexual scenes, how to continue valuing respect for the diversity and fluidity of sexual and gender identities, diversity that the secular state and the constitutionally defined right to health must protect.

The experience of public health has already taught us that having safe, effective, and adequate inputs for anything, be it vaccines, treatments, chemoprophylaxis, is not enough. Health vulnerabilities are created where human rights are disrespected and the practical wisdom of users is disregarded. On the other hand, contexts in which we identify vulnerabilities in health show challenges and opportunities for the construction of a more just, supportive, and happy society.
